# Investigating the dimensions of learning organizations questionnaire (DLOQ) in a Romanian private ophthalmology organization

**Published:** 2018

**Authors:** Consuela-Mădălina Gheorghe, Victor Lorin Purcărea, Iuliana-Raluca Gheorghe, Ovidiu Popa-Velea

**Affiliations:** * “Carol Davila” University of Medicine and Pharmacy, Bucharest, Romania

**Keywords:** DLOQ, Romanian private ophthalmology organization, Learning organization

## Abstract

Private ophthalmology organizations are knowledge-based institutions that need to adapt to changes from both external and internal environments. To ease the organization’s survival, a learning process is required at different levels: individual, team, group, and to the organization itself, triggering a learning organization (LO) transformation.

The aim of this research was to assess the relevance and efficiency of the Dimensions of the Learning Organization Questionnaire (DLOQ) in a private ophthalmology organization from Bucharest, Romania.

The DLOQ was translated from English into Romanian and administered to 113 nurses and physicians working in the private ophthalmology organization. The DLOQ includes the following dimensions: Continuous learning, Dialogue and inquiry, Team learning and collaboration, Embedded systems, Empowerment, Systems connections and Strategic Leadership. Data was analyzed using central tendency indicators, such as the mean and standard deviation for quantitative variables, as well as the frequency, for qualitative variables. To be able to determine the comparison between the DLOQ dimensions’ scores and the professions of the respondents, several Mann-Whitney U tests were performed. The DLOQ’s internal consistency and its measurement validity were assessed using the Cronbach’s alpha coefficient values and the Confirmatory Factor Analysis, respectively.

The findings of the DLOQ implementation revealed that it is generally suitable to be used in the ophthalmology context. However, among the DLOQ dimensions, the *Strategic Leadership* dimension had to be removed, due to cultural and socio-demographic factors. No significant variations across professions and dimensions were registered.

Although the DLOQ was developed for company settings, this study demonstrated that it could be successfully applied in health care as well. The DLOQ dimensions may provide valuable insights and understanding regarding the objective where further efforts should be directed. Also, through appropriate care management strategies, this instrument may contribute to the strengthening of the health care system, and particularly to the implementation of LO orientation in other medical specialties.

## Introduction

In a knowledge-based economy, the power of knowledge becomes a fundamental resource for organizations, as it preserves heritage and triggers challenging opportunities [**1**]. In the last decades, knowledge has become a very important factor in achieving a dynamic advantage in an environment defined by globalization, technical evolution, and demographic change [**2**,**3**]. This rapidly changing environment requires that organizations should constantly adapt in order to prosper, a valuable direction being represented by transforming the organization into a “learning organization” (LO) [**2**,**3**].

A LO is characterized by its capacity to adapt to its external environment, while in its internal environment, it implements a culture in which learning from challenges and mistakes is of great importance [**3**]. LOs are generally considered more functional and more efficient in any given background [**3**,**4**], however, this is not the case of Romanian health care system, described often as being the poorest and most inefficient in the European Union [**4**]. With costs per capita, which are almost seven times less than the European mean (353$ vs. 2.619$), and with one of the lowest densities of health care professionals in Europe (less than two physicians for 1.000 population, and less than four nurses for 1.000 population), Romania struggles to deliver efficient health care services and, at the same time, to decrease the mortality rates [**4**,**5**]. This lack of effectiveness in the functioning of public health services, largely due to their never-ending changes and inconsistent management, has steadily lead to the upsurge of founding private health care organizations [**6**]. From a management perspective, the private health care institutions have been organized so far both as hospitals with a wide range of specialties and in the shape of hospitals with a single specialty. 

In Romania, ophthalmology represents a field of great interest and in full development. According to the Romanian Institute of Statistics, in 2016 there were 38 private ophthalmology organizations and 467 specialized physicians working in these institutions [**7**]. The newest objective of Romanian private ophthalmology organizations is their transformation into LOs, even if this process is a difficult task, because of the variability in instruments used for determining the presence of LO principles or their levels of implementation. Moreover, many LO instruments have their own theoretical background and have been mainly developed and tested in high-income environments [**8**]. Consequently, there is a substantial need in practice for the establishing of a LO instrument that would be both valuable, irrespective of the underlying theoretical paradigm, and valid in low and middle-income contexts as well. In this sense, a plausible candidate is the Dimensions of the Learning Organization Questionnaire (DLOQ), developed by Marsick and Watkins, as it is, on one hand, the most researched and applied instrument for measuring the LO, and on the other hand, is described in literature as achieving scope, depth and reliability [**9**].

The aim of this study was to investigate the DLOQ instrument, in order to assess an in-depth view of its strengths and weaknesses in a Romanian private ophthalmology context. The key element of this research is that it brings forward practical evidence to validate the DLOQ for the clinical managerial use in Romania.

## Theoretical background

Most specialists consider learning as a process consisting in a sequence of activities based on knowledge acquisition, deeper understanding of situations, as well as improved performance [**10**]. According with the learning concept, LO, albeit difficult in defining [**11**], is characterized by a number of points: (a) a continuous learning process at different levels in an organization, namely individual, team or group levels; (b) emphasis on the creation and distribution of knowledge and information; (c) capacity of the organization to adapt to change, and (d) ability of the employees to improve the organizational performance by learning [**2**]. In line with these characteristics, the LO concept is focused on the basic elements of leadership, dialogue and inquiry, team learning, empowerment and organizational approval of processes and structures [**10**,**12**].

Specifically in health care, the implication of patient-centered delivery services and the advancement of technology have forced the medical staff to permanently upgrade their competences and knowledge through learning. These factors and the objective to ensure consistent quality of service have made health care organizations to consider themselves as LOs. Still, the real level of LO in these organizations needs to be further investigated and objectified. In this sense, literature pinpoints that, across different methods used in conceptualizing the construct, the most comprehensive instrument is The Dimensions of the Learning Organization Questionnaire (DLOQ) [**13**]. The DLOQ was designed to measure the learning organizational culture and intends to capture the employees’ perceptions regarding the seven dimensions included in the scale (**Table 1**). These dimensions measure the positive nature and cultural features of a supportive LO [**8**]. 

Currently, there are two versions of the DLOQ: a full one, which encompasses 43 measurement items and has been used as a diagnostic instrument for specialists who need a comprehensive assessment and information of the learning culture, in order to make strategic decisions, and an abridged one, comprising 21 items, used mainly for research purposes [**8**, **14**]. In this study, we used the 21-item version of DLOQ, because of its psychometric properties, ease of completion, and higher follow-up rates [**8**].

**Table 1:** Definitions of the DLOQ dimensions

Dimension	Definition
Continuous learning	Learning is embedded into work so people can learn on the job; opportunities are provided for ongoing education and growth.
Dialogue and inquiry	People gain productive reasoning skills to express their views and the capacity to listen and inquire into the views of others; the culture is changed to support questioning, feedback and experimentation.
Team learning and collaboration	Work is designed to use groups to access different methods of thinking; groups are expected to learn together and work together; collaboration is valued in the organizational culture and rewarded.
Embedded systems	Both high and low technology systems to share learning are created and integrated with work; access is provided, systems are maintained.
Empowerment	People are involved in setting, owning and implementing a joint vision; responsibility is distributed close to decision making so as people are motivated to learn what they are held accountable to do.
Systems connections	People are helped to see the effect of their work on the entire organization; people scan the environment and use information to adjust work practices; the organization is connected to the communities from its environment.
Strategic Leadership	Leaders support learning: leadership uses learning strategically for business results.
*Source: Leufven M, Vitrakoti R, Bergstrom A, Ashish KC, Malqvist M. Dimensions of Learning Organizations Questionnaire (DLOQ) in a low-resource health care setting in Nepal. Health Research Policy and Systems. 2015; 13(6),3*.	

## Material and Methods

• **Setting, sampling and participants**

The setting of the study consisted of a private ophthalmology organization from Bucharest, Romania, which offered a wide range of ophthalmological services (mainly surgical). The study design was cross-sectional. The collection of data was based on voluntary participation, by filling in a written consent form. The respondents were informed that all information was confidential and would be used for research purposes only. 

Questionnaires were distributed to 150 health care employees who met the following inclusion criteria: (a) physicians or nurses, with 1 to 20 years of employment in the private ophthalmology organization, and (b) no current or previous leading positions in the organization. 113 valid questionnaires were returned (this corresponding to a response rate of 75.3%).

• **Development of the research instrument**

The shorter version of the DLOQ with 21 items encompassed the following dimensions: Continuous learning (3 items), Dialogue and inquiry (3 items), Team learning and collaboration (3 items), Embedded systems (3 items), Empowerment (3 items), Systems connections (3 items) and Strategic leadership (3 items) [**15**,**16**,**18**]. The items included in each dimension were measured on 5-point Likert scales, ranging from 1-*Totally Disagree* to 5-*Totally Agree*.

Initially, a pilot test of the 21-item DLOQ original form was conducted on a number of 12 respondents. The results revealed that the participants did not have enough knowledge on leadership, so the *Strategic Leadership* dimension was removed. Next, the remaining 18-item DLOQ instrument was again tested on a sample of 20 respondents. The results indicated that the items in each dimension had satisfactory clarity and coherence. Thus, no further changes were made on the DLOQ instrument.

Finally, the questionnaire had two sections:

- the first section gathered demographic information about the participants (i.e., age, type of employment, gender, marital status, year of employment and position in the organization);

- the second section comprised the following DLOQ dimensions: Continuous learning (3 items), Dialogue and inquiry (3 items), Team learning and collaboration (3 items), Empowerment (3 items) and System connections (3 items).

• **Data analysis**

The collected data was subjected to descriptive analysis and factor analysis, using SPSS® version 20. The descriptive analysis consisted of measuring the mean (± standard deviation) of each item and dimension, as well as the frequency for the demographic information of the respondents. Further, Mann-Whitney U tests were performed, in order to determine comparisons between the responses offered by nurses and physicians. The factor analysis consisted of the Confirmatory Factor Analysis steps (CFA), and the Cronbach’s alpha coefficient, to evaluate the internal consistency of the DLOQ dimensions. A Principal Component Analysis (PCA) was employed to extract the major contributing factors. Varimax rotation was conducted, in order to identify the factor loadings. A factor loading greater than 0.40 was considered significant [**2**]. To assess the items’ internal consistency, zero-order correlation analysis and scale reliability tests were applied. A Cronbach’s alpha coefficient exceeding 0.70 was considered sufficient to assert internal consistency in the dimensions’ measurement.

## Findings

**1. Demographic data **

The participants were adult employees, with the mean age of 43.91 (±6.07). Most of the respondents were males (52.2%) and married (39.8%). The percentage of nurses and physicians taking part in the study was similar (47.8% vs. 52.2%). Also, the respondents were divided equally between those employed on a temporary vs. on a permanent basis (50.4% vs. 49.6%) (**Table 2**).

**Table 2:** Demographic data of the study participants

Variables	Number	%
Gender		
Female	54	47.8%
Male	59	52.2%
Position in the organization		
Physician	59	52.2%
Nurse	54	47.8%
Type of employment		
Temporarily employed	57	50.4%
Permanently employed	56	49.6%
Marital status		
Unmarried	32	28.3%
Married	45	39.8%
Divorced	36	31.9%
Period of employment in years		
≤ 1	6	5.3%
2-5	26	23%
6-10	36	31.9%
11-15	30	26.5%
16-20	15	13.3%

**2. Data resulting from the DLOQ instrument**

The descriptive statistics for all DLOQ items and dimensions are figured in **Tables 3** and **4**.

**Table 3:** Descriptive statistics for the items

Dimension	Items	Mean	Standard deviation
Continuous Learning	Q1	3.33	1.398
	Q2	3.29	1.178
	Q3	3.33	1.305
Dialogue and inquiry	Q4	3.37	1.174
	Q5	3.40	1.250
	Q6	3.33	1.168
Team learning and collaboration	Q7	3.46	1.267
	Q8	3.50	1.200
	Q9	3.51	1.135
Embedded systems	Q10	3.33	1.250
	Q11	3.24	1.331
	Q12	3.20	1.324
Empowerment	Q13	3.39	1.257
	Q14	3.19	1.243
	Q15	3.34	1.207
Systems connection	Q16	3.64	1.181
	Q17	3.37	1.248
	Q18	3.51	1.276

**Table 4:** Descriptive statistics for the DLOQ dimensions

Dimension	Mean	Standard deviation
Continuous Learning	9.95	3.380
Dialogue and inquiry	10.10	3.094
Team learning and collaboration	10.47	3.039
Embedded systems	9.77	3.396
Empowerment	9.91	3.278
Systems connection	10.52	3.157

**Fig. 1:**
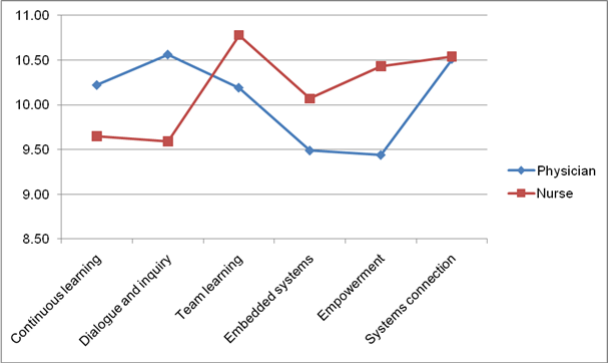
The scores of the DLOQ Dimensions as distributed by profession

The means of Q1 to Q18 ranged from 3.19 at Q14 (*My organization gives people control over the resources they need to accomplish their work*) to 3.64 at Q16 (*My organization encourages people to think from a global perspective*). In **Table 4**, the means of the dimensions ranged from 10.52 (at the dimension *Systems connections*) to 9.77 (at the dimension *Embedded systems*). Scores for the dimensions, distributed by profession, are displayed in **Fig. 1**. For physicians, the mean ranged from 9.44 (at the dimension *Empowerment*) to 10.56 (at the dimension *Dialogue and inquiry*). For the nurses, the mean ranged from 9.59 (at the dimension *Dialogue and inquiry*) to 10.78 (at the dimension *Team learning and collaboration*). 

A series of Mann-Whitney U tests were conducted to compare the scores between each of the professions and the items, as well as the dimensions. No significant differences were registered (p>0.05).

Next, the CFA was used, in order to confirm the construct validity of the DLOQ. The DLOQ measurement scale consisted of six dimensions, with a total of 18 items. The CFA outcome is illustrated in **Table 5** and it provides evidence that the DLOQ has high construct validity, because each statement has loading values greater than 0.80 and load properly on the initial designated dimension. Moreover, the Cronbach’s alpha coefficient values, depicted in **Table 5**, show, in their turn, a high internal consistency of the DLOQ scale (values>0.70).

**Table 5:** The factor loadings and the Cronbach’s alpha coefficients

Mean	Mean	Mean	Mean	Mean	Mean	Mean
Q1		0.850				
Q2		0.875				
Q3			0.865			
Q4				0.866		
Q5				0.819		
Q6				0.817		
Q7						0.815
Q8						0.829
Q9						0.869
Q10			0.848			
Q11			0.901			
Q12			0.854			
Q13	0.864					
Q14	0.875					
Q15	0.894					
Q16					0.848	
Q17					0.846	
Q18					0.841	
Cronbach’s alpha coefficient	0.860	0.838	0.839	0.825	0.811	0.794
*Legend: F1 = Empowerment; F2 = Continuous Learning; F3 = Embedded systems; F4 = Dialogue and inquiry; F5 = Systems connections; F6 = Team learning and collaboration*						

## Discussion

The objective of this study was to validate the DLOQ in a specific health care context, namely in a private ophthalmology organization. Our findings reveal that the applicability of DLOQ in the ophthalmology environment is satisfactory, although partially supported, given the fact that one dimension - *Strategic Leadership* - was removed. This may have been due to the different cultural backgrounds of respondents or of the different groups they are associated to, which are described in literature to be at the origin of intricate, nonlinear interactions [**19**]. On the background of a slow progress in promoting and developing health care leadership among the medical staff [**20**,**21**], health care providers may not have a clear picture about the importance of this construct and may not be prepared to provide it [**20**]. In addition, they have seldom career pathways, which stimulate them to actively engage in health care leadership roles and specific activities [**22**]. 

Further, our findings indicate that the respondents scored lower on the *Embedded systems* scale, with an overall score of 9.77 out of 15, suggesting a potential area of improvement. A potential explanation for this result lies in the mere definition of the underlying concept. As proposed by Marsick and Watkins [**9**], this dimension is typically understood as the property of the technology systems designed for sharing learning to be created and integrated in daily work, combined with the provision of access, so that these systems are maintained [**9**]. From the perspective of this definition, it is probable that, from a Romanian physician’s perspective, every private health care unit is properly equipped with the required technology and knowledge in order to provide the best medical care, so it comes as a common sense for them to perceive embedded systems as readily available.

The dimension that scored the overall highest value (10.52) was the one concerning *Systems connections*, which is described in literature as the ability of LOs to have healthy relationships with their physical, social, and cultural environments, and the support offered to their employees to see the impact of their work on the entire organization [**3**]. This finding may be attributed to the high concern of the studied organization for the implementation of efficient Corporate Social Responsibility strategies.

Despite that our results suggest DLOQ as a valuable instrument in measuring the LO principles in a health care context, further exploration of the usefulness of this and other instruments assessing the existence and characteristics of LO is needed. It is also important to combine this instrument with other forms of employees’ feedback assessment, such as mixed-method evaluative and research approaches, which would examine the detailed functional practices and outcomes of the DLOQ framework [**2**]. Such efforts can contribute in transforming health care providers into efficient leaders, able to influence the quality of patient care, the productivity, and performance of their teams and the strategic direction of their health care organizations [**23**]. On a long term, even if effective health care leadership is difficult to be evaluated and implemented in practice, it has substantial implications in daily care, by improving both the clinical outcomes in patients and the provider’s well-being at the workplace, thereby reducing the risk of burnout [**24**-**26**].

From a decisional perspective, our findings argue in favor of a shared clinical leadership, which distributes the leadership duties among individuals, instead of being promoted by only one individual [**20**]. In practice, managers of health care organizations should encourage the implementation of shared leadership, via teamwork, democracy and a flatter organizational structure. 

Additionally, the explicit promotion of leadership development curricula and training in medical education can contribute to a change in a durable change in the paradigm of functioning of health care organizations [**24**]. Obviously, health care leadership development in education should become intentional, and not only informal or implicit. 

**Limitations of the study**

Even if our study was among the first in Romania focusing on the LO concept and developed in a clinical setting, we are aware of some limitations:

- firstly, the DLOQ was translated into Romanian from English and back-translated by two independent experts. There is a likelihood that the translation, in some parts, failed to capture the semantic meanings of the items, due to the lack of appropriate term equivalencies or cultural misinterpretations;

- secondly, the sampling method has not accomplished the desired generalizability, as we applied the DLOQ in one private ophthalmology organization. Subsequent biases might have been derived from specific personal attitudes, job satisfaction and type of employment; 

- thirdly, the socio-cultural limitations of the DLOQ instrument, which was developed specifically for a Western high-income setting, may have missed to measure some other dimensions, specially those encountered in low and middle income countries. 

## Conclusions

Over the past two decades, literature data has steadily indicated three factors that are of core value in an organization’s survival and adaptability to the external forces: a supportive environment, concrete learning processes and practices, as well as reinforcement, through leadership behavior [**17**]. In this particular context, our study, focused specially on the second factor mentioned above, contributes to a more nuanced understanding of how the concept of LO is perceived by clinicians themselves. Specifically, gathered data confirmed that DLOQ is a valuable tool in measuring LO (still, without the dimension of *Strategic Leadership*). Further research should investigate if the DLOQ instrument can be successfully applied in other medical specialties, how exactly the characteristics of health care leadership relate to efficient patient care, and how LO principles can be effectively connected in practice to other basic concepts, such as internal marketing or organizational commitment. 
